# Inter- and intra-observer variability in Sonographic measurements of the cross-sectional diameters and area of the umbilical cord and its vessels during pregnancy

**DOI:** 10.1186/1742-4755-5-5

**Published:** 2008-10-15

**Authors:** Cristiane Barbieri, Jose G Cecatti, Carla E Souza, Emilio F Marussi, Jose V Costa

**Affiliations:** 1 Department of Obstetrics and Gynecology, School of Medical Sciences, University of Campinas – UNICAMP, Campinas, Brazil

## Abstract

**Background:**

The purpose of the study was to evaluate inter- and intra-observer variability in sonographic measurements of the cross-sectional area of the umbilical cord and the diameters of its vessels in low-risk pregnancies of 12 to 40 weeks of gestation.

**Methods:**

A prospective cross sectional study was performed in 221 pregnant women at different gestational ages. Measurements were carried out also by a second observer to evaluate inter-observer variability and repeated once again by the first observer to assess intra-observer variability. The linear correlation between the measurements (Spearman's coefficient of correlation) and their reliability through the intraclass correlation coefficient (ICC), the Cronbach's alpha coefficient and the limits of agreement proposed by Bland and Altman were evaluated.

**Results:**

The results showed that inter-observer and intra-observer variability did not show any significant difference between examiners. A good linear correlation between the measurements and reliability was obtained, with values of R, ICC and Cronbach's alpha all above the standard limits.

**Conclusion:**

It is possible to conclude that inter- and intra-observer variability in the measurements of the umbilical cord and its vessels was small; their reliability and agreement were good.

## Background

Ultrasonography has been known for quite some time to be a useful tool for the detection of congenital abnormalities, in the diagnosis of multiple pregnancies, in locating the placenta, evaluating fetal growth, and in identifying pregnant women at risk of postmaturity or intrauterine growth restriction [1,2]. More recently, morphology of the umbilical cord, including its diameter and the amount of Wharton's jelly, have been associated with adverse perinatal events, such as preeclampsia [3], gestational *diabetes mellitus *[4], intrauterine growth restriction [5], small-for-gestational-age fetuses [6,7], fetal distress during labor and indication for Cesarean delivery [8]. Bruch et al. [9] also showed that the areas of the umbilical cord and vessels are smaller in fetuses with a diagnosis of intrauterine growth restriction. The same occurs in pregnant women with chronic hypertension and preeclampsia [10]. Under these conditions, the reduction in the vein lumen area would lead to an increase in resistance to blood flow and a consequent remodeling of fetal-placental hemodynamics.

Using an ultrasound scanner with high image resolution and amplification that permits adequate vision of the vessels and their contours, the umbilical cord is easily identified even in the initial stages of pregnancy, permitting early detection of any changes in its thickness [11]. Therefore, this could theoretically, become another useful tool for the prognostic selection of cases in which associated adverse effects are more likely to develop, particularly in the case of high risk pregnancies [12].

Nevertheless, sonographic measurement of the diameter of the umbilical cord and its vessels is yet to become routine practice in obstetrics [13]. Although there is no clear explanation for this, some of the difficulties that are presumed to be the principal determining factors in the technique not having yet been incorporated into routine healthcare during pregnancy include the absence of a universally accepted reference curve for these measurements, the lack of effective validation of these measurements in different populations, and possible technical difficulties in performing the measurements [14]. This present study is part of a larger study designed to construct a reference curve of measurements of the cross-sectional area of the umbilical cord in low-risk pregnancies. The objective of this study was to evaluate inter- and intra-observer variability in sonographic measurements of the diameter of the umbilical artery, the umbilical vein, the umbilical cord and the cross-sectional area of the umbilical cord.

## Methods

This was a prospective cross sectional study to compare the variability in sonographic measurements of the umbilical cord and its vessels when carried out by the same evaluator or by different evaluators. It was estimated that 214 exams would be necessary to assess inter and intra-observer variability of these measurements, considering a type I error of 0.05 and a power of 80%, without considering gestational age. A total of 221 patients with low risk pregnancies of gestational ages ranging between 12 and 40 weeks, who had been referred for routine ultrasonography, were evaluated once between June 2005 and December 2006.

Inclusion criteria comprised: single gestation, live fetus, gestational age previously established by the date of last menstrual period (LMP) if reliable or ultrasonography carried out in the first trimester, unruptured membranes, and normal amniotic fluid index [15]. Patients with *diabetes mellitus*, gestational diabetes, hypertension of any etiology, fetal malformations, oligoamnios or polyhydramnios, fetuses with signs of intrauterine growth restriction (estimated fetal weight below the 10^th ^percentile) or signs of fetal macrosomia (estimated fetal weight above the 90^th ^percentile) and abnormalities in the morphology of the umbilical cord up to the moment of the ultrasound exam, were excluded from the study.

A Power Vision 6000 ultrasound scanner (model SSA-370^® ^from Toshiba Medical Systems, Tokyo, Japan) or a Voluson 730 PRO^® ^scanner (General Electric Medical Systems, Milwaukee, Wisconsin, USA) with a 3.5 mHz transabdominal convex transducer, adopted as the standard equipment for obstetrical examinations, were used for all the ultrasonography scans carried out in this study.

Patients were submitted to routine ultrasonography in the semi-seated position. Parameters for the estimation of fetal weight were measured (biparietal diameter, head and abdominal circumferences and femur length), and the amniotic fluid index, location and grade of the placenta, and fetal position were evaluated. Patients who fulfilled the inclusion criteria were then informed about the study and any queries were answered, after which they were invited to participate in the study. All patients who agreed to participate signed an informed consent form. The research protocol was previously approved by the Institutional Review Board of the institution (approval #268/2005). Each women participating in the study signed an informed consent prior to be enrolled, following all recommendations of the Declaration of Helsinki.

**Figure 1:**
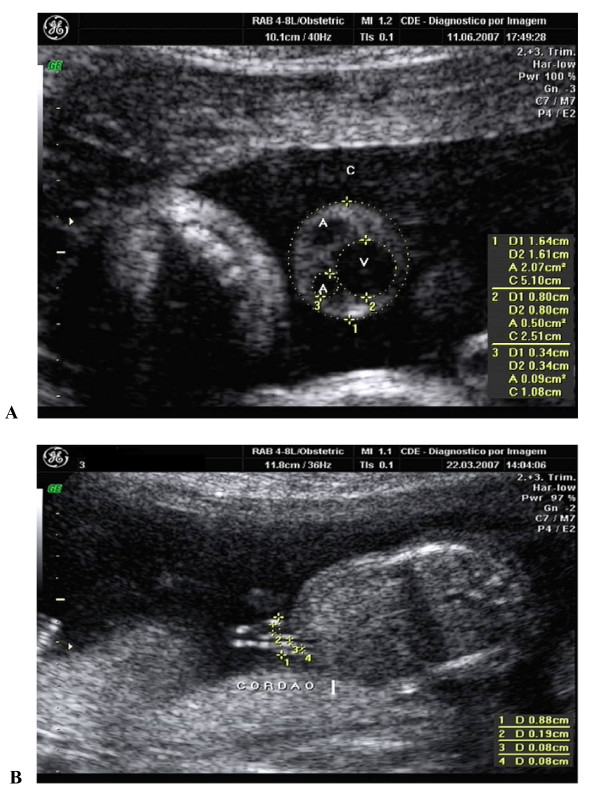
**Ultrasonographic measurement A.** of the cross-sectional area of the umbilical cord (C), the diameter of the umbilical vein (V) and the umbilical artery (A); and B. of the longitudinal section of the umbilical cord (between 12 and 14 weeks).

Next, the diameter and the cross-sectional area of the umbilical cord and the diameters of its vessels (arteries and vein) were measured in all women after 14 weeks of gestation. Measurements were carried out in a cross-sectional plane to the cord, adjacent to its insertion into the fetal abdominal wall, within a maximum distance of 2.0 cm, using the elliptical calipers of the ultrasound scanners, at the outer borders of the cord and at the inner borders of the vessels (umbilical vein and arteries), as shown in Figure 1A (method used by Ghezzi et al. [16]). In the case of pregnancies of 12–14 weeks of gestational age, cranial-caudal length was measured during a period of fetal rest in a longitudinal section. In some cases, a 7.5 mHz endovaginal probe was also used. The diameters of the cord and its internal vessels were measured in a free loop of cord immediately adjacent to its insertion into the fetal abdominal wall, placing the markers at its outer borders and, with maximum image magnification, along its longitudinal axis, according to the technique described by Ghezzi et al. [17] (Figure 1B). Only one artery was measured because there is an agreement that the variability between them is low.

To evaluate inter- and intraobserver variability, measurements of the umbilical cord (area of the cord, diameters of the cord, vein and arteries) were initially made always by the same first examiner (CB, 5 years experience in ultrasound in Obstetrics and Gynecology, with national certification). Next, another second examiner (CES, 3 years experience in ultrasound in Obstetrics and Gynecology, with national certification), previously informed about the nature of the study, took the same measurements from another image obtained with no knowledge of the previous results. Finally, the same first examiner repeated the measurements again according to the criteria established above. All measurements were independently recorded and photographed.

To evaluate inter- and intra-observer variability of the measurements of the cross-sectional area of the umbilical cord and the diameters of its vessels, the mean difference in the measurements of the two observers was first calculated, as well as their respective standard deviations (inter: measurement 2 – measurement 1; intra: measurement 3 – measurement 1), and 95% confidence intervals (95%CI) with the normality of the data checked with the Kolmogorov Smirnoff test and the statistical significance of these mean differences evaluated using the Mann-Whitney non-parametric test. P-values < 0.05 were considered statistically significant. Next, the following analyses were performed: the linear correlation between measurements (Spearman's coefficient of correlation), with values >0.7 being considered indicative of good agreement [18]; the reliability of the measurements evaluated by their reproducibility (intraclass coefficient of correlation – ICC) [19], with values >0.8 being considered as excellent [20]; and internal consistency (Cronbach's alpha), with values >0.8 being considered indicative of good reliability [21,22]. Finally the 95% agreement limits were graphically evaluated according to the method proposed by Bland and Altman [23], using proportions of the difference between both measurements in relation to the mean value.

## Results

The principal characteristics of the 221 pregnant women evaluated are shown in Table 1. Most of the women were white, and 46.6% were nulliparous. Forty-four percent were between 20 and 29 years of age. The ultrasonographic evaluations were carried out at different gestational ages.

**Table 1:** Characteristics of study population

Characteristics	n	%
Nulliparous	103	46.6
Previous abortion	27	12.2
At least one living child	118	53.4
		
White	168	76.0
		
Age (years)		
14 – 19	21	9.5
20 – 29	98	44.3
30 – 39	85	38.5
40 – 45	17	7.7
		
Gestational age (weeks)		
14 – 16	7	3.2
17 – 21	23	10.4
22 – 24	23	10.4
25 – 28	43	19.5
29 – 32	50	22.6
33 – 36	30	13.6
37 – 40	45	20.4
		
Total	221	

**Figure 2:**
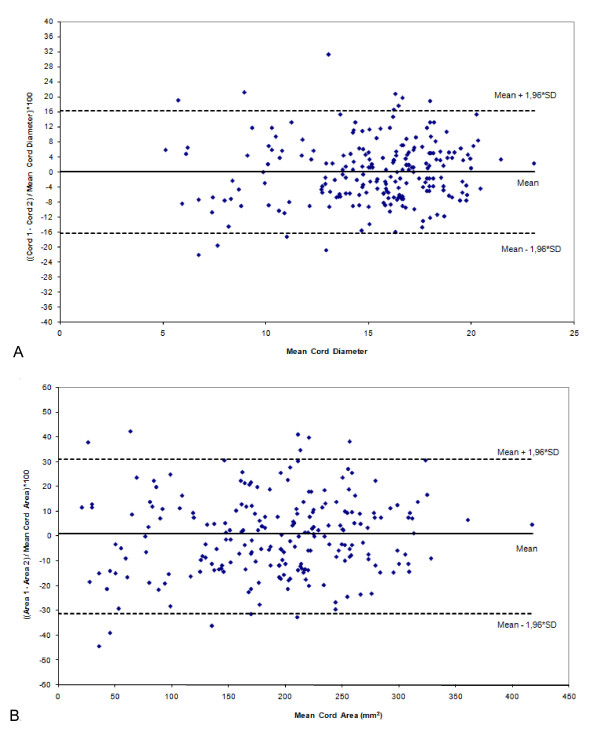
**Inter-observer agreement by plotting the mean differences against the mean values (method of Bland & Altman) for the following measurements:** A. Diameter of the umbilical cord (cord_1 _and cord_2 _are respectively the measurements of observer 1 and 2); and B. diameter of the area of the umbilical cord (area_1 _and area_2 _are respectively the measurements of observer 1 and 2).

Comparison between the measurements of the first and second evaluators (interobserver variability) indicated a trend to slightly overestimate the diameter of the umbilical vein, and the umbilical cord and its area, and to underestimate the diameter of the umbilical artery (Table 2). However, these differences were not statistically significant. The difference between the measurements obtained by the two different examiners was found to be dispersed around the mean, with no clear trend towards over- or underestimation by either one of these examiners, as graphically seen through the 95% agreement limits of Bland and Altman (Figure 2).

**Table 2:** Inter-observer variability in the ultrasonographic measurements of the umbilical cord (n = 221)

Characteristics	Mean Difference (SD)	95%CI	p*	R	ICC	Crombach's alpha
Diameter of the cord (mm)	0.024 (1.208)	(-0.136; 0.185)	0.896	0.90	0.94	0.97
Diameter of the artery (mm)	-0.057 (0.677)	(-0.147; 0.032)	0.728	0.79	0.83	0.91
Diameter of the vein (mm)	0.045 (0.907)	(-0.075; 0.166)	0.800	0.90	0.91	0.95
Area of the cord (mm^2^)	1.088 (30.120)	(-2.905; 5.081)	0.903	0.90	0.92	0.96

* Mann-Whitney's non parametric test.

R: Spearman's correlation coefficient (p < 0.0001).

ICC: intraclass correlation coefficient (internal consistency).

**Figure 3:**
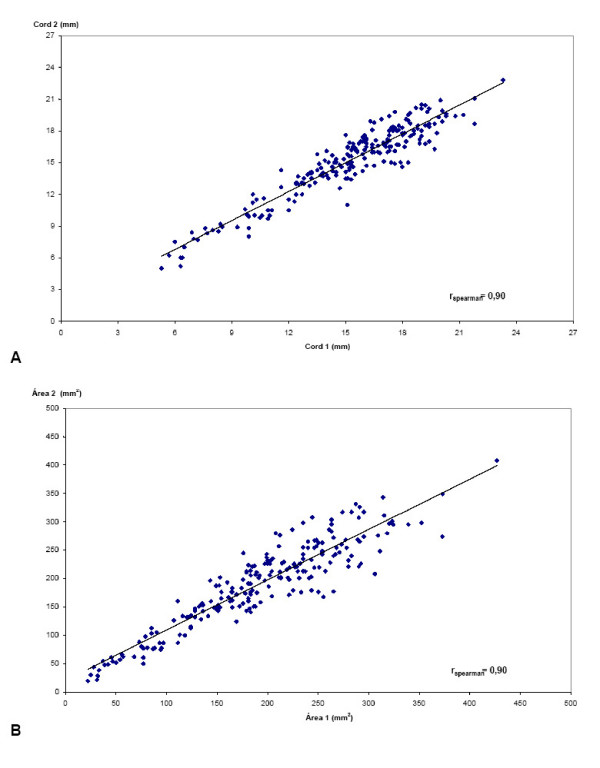
**Linear correlation (r = correlation coefficient of Spearman) of the inter-observer ultra-sonographic measurements:** A. of the diameter of the umbilical cord (cord_1 _and cord_2 _are respectively the measurements of observer 1 and 2); and B. of the cross-sectional area of the umbilical cord (area_1 _and area_2 _are respectively the measurements of observer 1 and 2).

The linear correlation between the measurements (Spearman's coefficient of correlation), their reliability (intraclass correlation coefficient – ICC) and internal consistency (Cronbach's alpha) were significantly high for all the measurements, being < 0.9 only in the case of the diameter of the umbilical artery (Table 2). Figure 3 illustrates the linear correlation between the interobserver measurements of the diameter and area of the cord.

**Figure 4:**
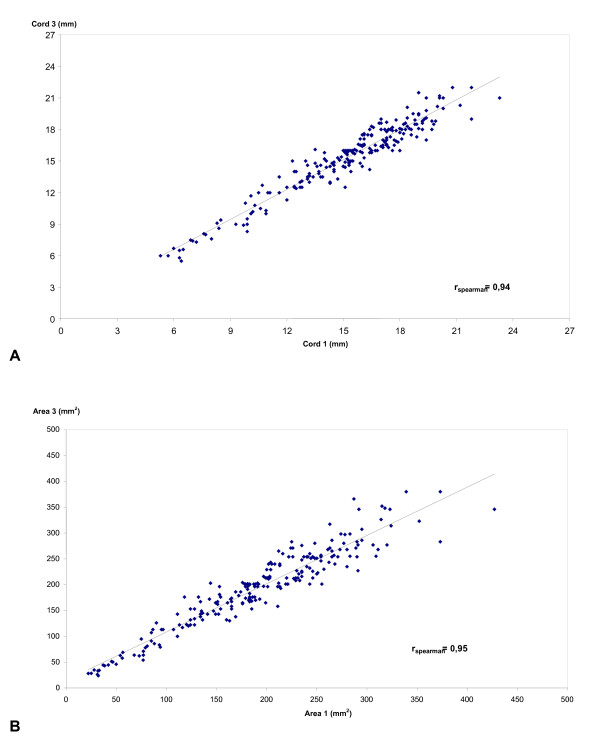
**Linear correlation (r = correlation coefficient of Spearman) of the intra-observer ultrasonographic measurements:** A. of the diameter of the umbilical cord (cord_1 _and cord_3 _are respectively the first and second measurements of observer 1); and B. of the cross-sectional area of the umbilical cord (area_1 _and area_3 _are respectively the first and second measurements of observer 1).

In the comparison between the two sets of measurements carried out by the first evaluator (intraobserver variability), there was a trend towards underestimation of the diameters of the cord, the artery and the area of the cord, with a small overestimation in the diameter of the umbilical vein. Nevertheless, again none of these differences was statistically significant. The linear correlation between the measurements, their reliability and internal consistency were significantly high for all the measurements, being < 0.9 only in the case of the diameter of the artery (Table 3). The linear correlations between the intraobserver measurements for the parameters of the diameter and area of the cord are illustrated in Figure 4.

**Table 3:** Intra-observer variability in the ultrasonographic measurements of the umbilical cord (n = 221)

Characteristics	Mean Difference (SD)	(95%CI)	p*	R	ICC	Crombach's alpha
Diameter of the cord (mm)	-0.120 (0.960)	(-0.248; 0.007)	0.734	0.95	0.96	0.98
Diameter of the artery (mm)	-0.137 (0.576)	(-0.213; -0.06)	0.332	0.86	0.88	0.94
Diameter of the vein (mm)	0.011 (0.763)	(-0.09; 0.113)	0.917	0.92	0.94	0.97
Area of the cord (mm^2^)	-2.500 (24.390)	(-5.74; 0.73)	0.732	0.95	0.95	0.97

* Mann-Whitney's non parametric test.

R: Spearman's correlation coefficient (p < 0.0001).

ICC: intraclass correlation coefficient (internal consistency)

## Discussion

The objective of using two different investigators to measure the cross-sectional area of the umbilical cord and the diameter of its internal vessels was to determine the precision of the method for use as an early screening tool for the detection of abnormalities that could be harmful to the fetus or the pregnancy. Reliability, reproducibility and precision are terms used to describe the extent to which the measurements of a stable phenomenon, repeated by different persons or instruments at different times or in different places, achieve similar results [22]. This evaluation is fundamental in assuring the predictive value of a measurement.

In the present study, in pregnancies of 12–14 weeks of gestational age, the measurements were carried out on a longitudinal section due to the greater difficulty in obtaining images of the cross-sectional area of the cord, while in the remaining women measurements were performed on a transverse section. This may represent a limitation to the study and consequently in its results due to the different techniques used in evaluating different gestational ages. Historically, this procedure was first reported in 1994 in a study carried out by Weissman et al. [13] in which the diameters of the umbilical artery, vein and cord, and the surface area of Wharton's jelly were measured between 8 and 42 weeks of pregnancy. For this evaluation, a longitudinal section of the umbilical cord close to its point of insertion into the fetal abdominal wall was used, since there was no difference between the measurements carried out using this section compared to the diameters measured in a transversal section of the cord; in addition, it provided better visualization in early pregnancies. Moreover, the diameters of the artery and the umbilical vein were measured only after 14 weeks of pregnancy.

Some years later, another study used the cross-sectional area of the umbilical cord to construct a normality curve of the diameters of the umbilical cord and its vessels in relation to fetal size [24]. According to these investigators, the cross-sectional area of the cord is a more reliable parameter, since the measurement of the diameter of the cord is influenced by the amount of Wharton's jelly. Moreover, the cross-section of the umbilical cord is not precisely circular, and this may lead to a slight underestimation in measurement. Recently, Togni et al. [25] also established normality curves using the cross-sectional areas of the umbilical cord and its vessels and the quantity of Wharton's jelly, and correlated them with fetal anthropometric parameters in low risk pregnancies of 24–39 weeks.

In the comparison of the umbilical cord measurements carried out by the different examiners, the present study shows that these differences were not statistically significant at all. The choice of each individual examiner with respect to the part of the cord in which to carry out the measurements (while respecting the standard distance of a maximum of 2.0 cm from the insertion of the umbilical cord into the fetal abdomen), and the presence of coiling along the cord may partially explain these differences. It should also be remembered that up to 40 coils may be present in the umbilical cord as its length increases with gestational age [26]. If the examiners randomly select the best transversal section in which to carry out their measurements within the standard distance from the umbilical insertion but in different locations within the coils, small variations in measurements would be expected.

In the case of the umbilical arteries, the diameter of only one artery was measured, each examiner selecting the one in which the contours were more visible. Generally, the umbilical arteries have similar lumen diameters; however, it is known that in around 0.7 to 1.4% of cases one of the umbilical arteries is smaller than the other [27]. Differences of around 1–3 mm have also been reported in their diameters [28], leading to differences in blood flow parameters and greater resistance in the vessel with lower caliber [29]. This may also contribute towards the differences found in this measurement.

On the other hand, in the evaluation of the differences obtained in the measurements of the vessels of the umbilical cord carried out by the same examiner, the small variations detected were not statistically significant. Spearman's correlation coefficient indicated good agreement between the measurements carried out by the different examiners for all the parameters studied, both with respect to inter- and intraobserver variability, thereby allowing us to assume that these measurements may be safely carried out by different examiners at different times in different locations. Although this study used a 3.5 MHz transabdominal probe for the measurements, similar results have been described using higher frequency probes what would improve the resolution of the images [16].

Ultrasonographic findings of abnormalities in the umbilical cord may be associated with fetal or chromosomic abnormalities, intrauterine fetal growth restriction and other pathological conditions related to an increase in fetal and neonatal morbidity and mortality [3,5,7]. Early detection of these changes may be important for maternal and fetal prognosis, but this was not one of the objectives of the present study. First, standards that would be valid for the reference population have to be established for these measurements. The ability of these standards to accurately predict conditions apparently associated with abnormalities of the umbilical cord must be validated [30].

Many of these findings are not isolated. For this reason, careful evaluation of the umbilical cord by measuring its vessels and the umbilical cord itself throughout the different phases of pregnancy may become a routine part of obstetrical care [31], and should not be restricted to detecting the number of umbilical vessels, the presence of cysts and Doppler evaluation of blood flow, as is current practice [32]. This will permit a qualitative evolution in perinatal care even during pregnancy by identifying those cases with a greater probability of developing maternal and fetal neonatal complications so that surveillance may be improved and prophylactic or therapeutic measures may be instituted at an earlier stage. If future studies confirm the predictive capability of abnormalities in these measurements for the various associated pathological conditions, the present study will have contributed towards demonstrating that these measurements are technically reproducible.

## Abbreviations

ICC: intraclass correlation coefficient; LMP: last menstrual period; R: Coefficient of correlation.

## Competing interests

The authors declare that they have no competing interests.

## Authors' contributions

CB and JGC participated in all steps of the study, including research planning, data collection, analysis and writing the manuscript. CES participated enrolling patients and performing ultrasound exams. EFM participated in the project planning and review of the manuscript. JVC was responsible for statistical analysis. All authors gave suggestions, read the manuscript carefully, fully agreed on its content and approved its final version.
